# Prevalence and Income-Related Disparities in Thirdhand Smoke Exposure to Children

**DOI:** 10.1001/jamanetworkopen.2021.47184

**Published:** 2022-02-07

**Authors:** Georg E. Matt, Ashley L. Merianos, Penelope J. E. Quintana, Eunha Hoh, Nathan G. Dodder, E. Melinda Mahabee-Gittens

**Affiliations:** 1 Department of Psychology, San Diego State University, San Diego, California; 2 School of Human Services, University of Cincinnati, Cincinnati, Ohio; 3 School of Public Health, San Diego State University, San Diego, California; 4 Division of Emergency Medicine, Cincinnati Children’s Hospital Medical Center, Cincinnati, Ohio; 5 Department of Pediatrics, University of Cincinnati College of Medicine, Cincinnati, Ohio

## Abstract

This cross-sectional study examines the prevalence of and income-related disparities associated with exposure to thirdhand smoke among children.

## Introduction

Thirdhand smoke (THS) is the residue that lingers on surfaces and in dust in environments where tobacco was used.^1,2^ Children face greater risks from THS exposure than adults because of more time indoors, frequent hand-to-mouth behaviors, high intake relative to body weight, immature immune systems, and developing organs.^2,3^ In this study, we estimate the proportion of children younger than 12 years who are exposed to THS in the absence of secondhand smoke and examine factors associated with THS exposure.

## Methods

We conducted a cross-sectional study wherein we screened children (younger than 12 years) seeking emergency care (n = 269) and the children of employees (n = 235) at Cincinnati Children’s Hospital Medical Center between February 2020 and May 2021. With approval from the hospital’s institutional review board, we followed Strengthening the Reporting of Observational Studies in Epidemiology (STROBE) reporting guideline for observational studies. Our primary outcome measure was field-blank-corrected nicotine levels on children’s hands as a THS marker. Written informed consent was obtained from parents, and written assent was obtained on children older than 11 years. After obtaining informed consent, we interviewed parents and wiped the palmar and volar surfaces of all fingers on children’s dominant hand with prescreened cotton rounds. We considered children protected from exposure to tobacco products if no household member smoked or vaped, smoking and vaping were banned in homes and cars, and there was no contact with tobacco users within the previous week. Parents self-reported their and their child’s race and ethnicity. Hand wipe samples were analyzed for nicotine using isotope-dilution liquid chromatography-tandem mass spectrometry.^3,4^ We used linear regression models to examine variables associated with hand nicotine (log-transformed). The type I error rate for *t* tests and *F* tests was 5%, and all tests were 2-sided. Analyses were performed with Stata, version 17 (StataCorp LLC).

## Results

The Table shows sociodemographic details, tobacco product use, and child tobacco smoke exposure. For the total sample of 504 children, 263 (52.2%) were boys and 241 (47.8%) were girls; 488 (97.0%) were non-Hispanic and 15 (3.0%) were Hispanic; 122 (24.2%) were Black or African American, 354 (70.2%) were White, 18 (3.6%) were of more than 1 race, and 10 (2.0%) were of other race (including Asian or American Indian or Alaska Native); and the mean (SD) age was 5.7 (3.3) years. Nicotine was detected on the hands of 189 of 193 children (97.9%) reportedly exposed (geometric mean [GeoMean], 21.8 ng/wipe; 95% CI, 16.5-28.7 ng/wipe) and on the hands of 296 of 311 children (95.2%) reportedly protected from exposure to tobacco products (GeoMean, 2.9 ng/wipe; 95% CI, 2.5-3.4 ng/wipe). In multivariable linear regression models, child age, family income, parent tobacco use, home smoking rules, and the number of tobacco users with whom a child had contact were significantly associated with hand nicotine (adjusted *R*^2^ = 0.63, *F*_20,483_ = 43.85, *P* < .001). Children aged 2 to 4 years, from low-income homes, whose parents used any tobacco products, with multiple contacts with tobacco users, and without complete smoking bans had the highest hand nicotine levels (Table). Children from lower-income families benefited significantly more from tobacco protections compared with children from higher-income homes (Figure) (*F*_6,487_ = 4.47, *P* < .001).

**Table.** Sociodemographic Characteristics, Tobacco Product Use, and Exposure of Children Protected From and Exposed to Tobacco Products

Characteristic	No. (%)			Linear regression model of hand nicotine, β̂ (SE)
	Total sample (N = 504)	Exposed to tobacco smoke (n = 193)	Protected from exposure to tobacco smoke (n = 311)	
Hand nicotine				
% >LOQ (95% CI)	96.2 (94.2-97.7)	97.9 (94.8-99.4)	95.2 (92.2-97.3)	NA
Geometric mean (95% CI), ng/wipe	6.7 (5.7-7.9)	21.8 (16.5-28.7)	2.9 (2.5-3.4)	NA
Median (IQR) [range], ng/wipe	3.6 (1.3-15.5) [0-2341.6]	20.0 (3.5-97.0) [0-2341.6]	2.4 (1.0-5.7) [0-233.1]	NA
Child age, y^a^				
0-1	88 (17.5)	39 (20.2)	49 (15.8)	−0.141 (0.060)^a^
2-4	136 (27.0)	55 (28.5)	81 (26.1)	[Reference]
5-7	133 (26.4)	51 (26.4)	82 (26.4)	−0.022 (0.054)
8-11	147 (29.2)	48 (24.9)	99 (31.8)	−0.105 (0.053)^a^
Child sex				
Male	263 (52.2)	100 (51.8)	163 (52.4)	[Reference]
Female	241 (47.8)	93 (48.2)	148 (47.6)	−0.055 (0.040)
Parent ethnicity				
Non-Hispanic	488 (97.0)	188 (97.9)	300 (96.5)	[Reference]
Hispanic	15 (3.0)	4 (2.1)	11 (3.5)	−0.002 (0.118)
Parent race				
Black or African American	122 (24.2)	78 (40.4)	44 (14.2)	0.093 (0.063)
White	354 (70.2)	107 (55.4)	247 (79.4)	[Reference]
More than 1	18 (3.6)	6 (3.1)	12 (3.9)	−0.130 (0.111)
Other^b^	10 (2.0)	2 (1.0)	8 (2.6)	−0.063 (0.141)
Parent education				
Less than high school	17 (3.4)	13 (6.7)	4 (1.3)	[Reference]
High school graduate	85 (16.9)	61 (31.6)	24 (7.7)	−0.007 (0.120)
Vocational or technical	19 (3.8)	9 (4.7)	10 (3.2)	−0.081 (0.158)
Some college	78 (15.5)	47 (24.4)	31 (10.0)	−0.088 (0.129)
College graduate	148 (29.4)	42 (21.8)	106 (34.1)	−0.204 (0.137)
Postgraduate	157 (31.2)	21 (10.9)	136 (43.7)	−0.112 (0.141)
Parent income, $^c^				
≤15 000	104 (20.6)	81 (42.0)	23 (7.4)	[Reference]
15 001-30 000	50 (9.9)	31 (16.1)	19 (6.1)	−0.213 (0.087)^a^
30 001-50 000	44 (8.7)	22 (11.4)	22 (7.1)	−0.380 (0.095)^c^
50 001-75 000	54 (10.7)	15 (7.8)	39 (12.5)	−0.366 (0.106)^c^
75 001-90 000	44 (8.7)	12 (6.2)	32 (10.3)	−0.342 (0.112)^c^
90 001-120 000	65 (12.9)	12 (6.2)	53 (17.0)	−0.402 (0.110)^c^
>120 000	143 (28.4)	20 (10.4)	123 (39.6)	−0.456 (0.105)^c^
Type of home				
Single family	382 (75.8)	112 (58.0)	270 (86.8)	[Reference]
Multiunit	68 (13.5)	47 (24.4)	21 (6.8)	0.087 (0.068)
Multifamily	54 (10.7)	34 (17.6)	20 (6.2)	−0.041 (0.073)
Parents’ tobacco product use^c^				
None	426 (84.5)	115 (59.6)	311 (100)	[Reference]
Cigarettes only	41 (8.1)	41 (21.2)	0	0.751 (0.104)^c^
Cigars only	19 (3.8)	19 (9.8)	0	0.287 (0.126)^a^
eCigarettes only	4 (0.8)	4 (2.1)	0	1.079 (0.232)^c^
Dual- or poly-use	14 (2.8)	14 (7.3)	0	0.316 (0.151)^a^
Home smoking rules^c^				
Complete ban	405 (80.4)	94 (48.7)	311 (100)	[Reference]
Allowed anywhere	34 (6.8)	34 (17.6)	0	0.486 (0.097)^c^
Usually allowed	26 (5.2)	26 (13.5)	0	0.237 (0.124)
Usually not allowed	17 (3.4)	17 (8.8)	0	0.495 (0.125)^c^
Only certain people	22 (4.4)	22 (11.1)	0	0.297 (0.118)^a^
Child contact with tobacco product users, No. of users^a^				
0	334 (66.3)	23 (11.9)	311 (100)	[Reference]
1-2	142 (28.2)	142 (73.6)	0	0.091 (0.055)
3-4	23 (4.6)	23 (11.9)	0	0.228 (0.108)^a^
≥5	5 (1.0)	5 (2.6)	0	0.419 (0.205)^a^

Abbreviations: β̂, partial regression coefficient; LOQ, level of quantitation (0.30 ng/wipe).

^a^ *P* < .05.

^b^ Other includes 7 Alaska Native or American Indian and 3 Asian participants.

^c^ *P* < .01.

**Figure.**
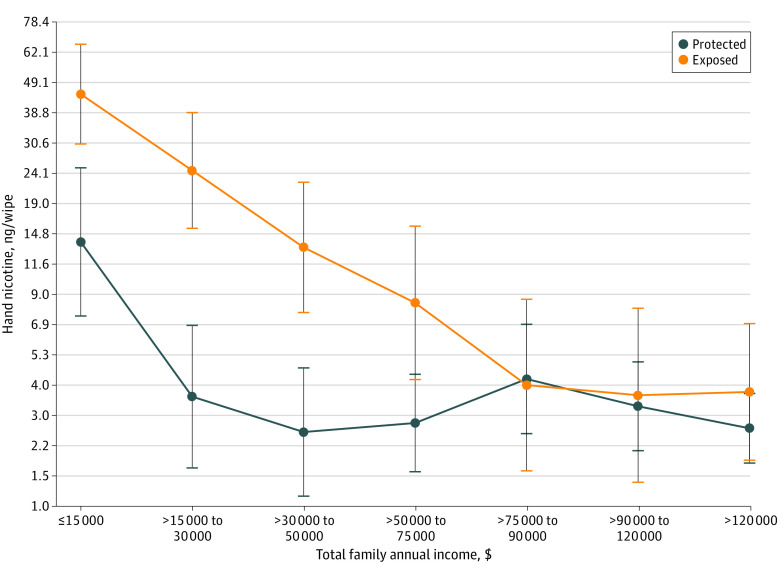
Mean Hand Nicotine Levels of Children Protected From and Exposed to Tobacco Smoke Products Geometric means and 95% CIs of child hand nicotine levels (ng/wipe) by different family income for children protected from (n = 311) and exposed to (n = 193) tobacco products.

Examining only children protected from tobacco products (n = 311), parent race and family income accounted for 27% of variance in hand nicotine (adjusted *R*^2^ = 0.24; *F*_10,300_ = 10.98, *P* < .001). The Figure indicates that among children believed to be protected, those from the lowest (≤$15 000; GeoMean, 14.2 ng/wipe; 95% CI, 8.7-22.7 ng/wipe) and second-lowest ($15 001-$30 000; GeoMean, 4.2 ng/wipe; 95% CI, 2.4-6.7 ng/wipe) annual income levels had 5.7 times and 1.7 times higher levels of hand nicotine, respectively, than children from families with incomes greater than $30 000 (GeoMean, 2.4 ng/wipe; 95% CI, 2.1-2.8 ng/wipe). Children of Black parents had higher nicotine levels (GeoMean, 4.9 ng/wipe; 95% CI, 3.2-7.2 ng/wipe) than children of White parents (GeoMean, 2.8 ng/wipe; 95% CI, 2.3-3.2 ng/wipe; *F*_1,300_ = 5.48; *P* = .02) or multiracial parents (GeoMean, 1.4 ng/wipe; 95% CI, 0.4-3.0 ng/wipe; *F*_1,300_ = 8.88; *P* = .003).

## Discussion

Although this cross-sectional study examined a convenience sample from 1 children’s hospital, the high prevalence of THS exposure among all children younger than 12 years is concerning, because there is no safe level of exposure to tobacco smoke toxicants.^5^ While parents’ efforts to protect their children from tobacco smoke pollutants did not fully prevent exposure, they reduced the magnitude of exposure by a mean of 86%. The association between income and hand nicotine among protected and unprotected children, independent of other variables, points to a troubling potential role of income-related disparities, such as housing type and quality, in THS exposure. Decades of permissive smoking policies have created significant THS reservoirs in many indoor environments. Thirdhand smoke can persist at stable levels over extended periods, creating conditions for chronic dermal, ingestion, and inhalation exposure to harmful THS constituents (eg, nicotine, tobacco-specific nitrosamines).^2,6^ Implementation of smoking bans, exposure screening, and THS remediation in homes between changes in occupants are needed to help protect children from THS.
